# Dental Radiology and Obscure Infections

**Published:** 1919-09

**Authors:** Fred S. O’Hara


### DENTAL RADIOLOGY AND OBSCURE INFECTIONS

BY FRED S. O'HARA, M.D.

Of exceeding importance was the position of the liaison-officer "over there." Over here, the connecting link between the physician and the dentist is analogous to that of the officer just mentioned and is filled only by the roentgenologist.

In those good old days (now visible only through the dust that we have raised by our high-powered thinking apparatus as it sped down the highway of years), "rheumatiz" and "neuralgy" were carefully and patiently treated, through years of time, with powder, pill, and potion. "Yarbs" were brewed *ad nauseam;* porous-plaster emporiums worked over-time to fill the demand; and wintergreen trees sobbed away their innocent lives in the knowledge that they would be early called upon to fill the emptiness in the gaultheria receptacles.

The doctor that could introduce the greatest number of ingredients in one bottleful of dope was the commander in the antirheumatic phalanx, and all sought that famous prescription wherein could be tasted everything from stewed onions to broiled rubber sheeting. Children had seven kinds of hell whipped out of them to cure the "growing pains," under the impression, evidently, that counter-irritation was worth its weight in porter-house steaks.

### A PROPHECY OF A NEW ORDER

Strange to relate, when grandma bade farewell to the last of her snaggy teeth and assumed the role of chief engineer to a full complement of artificial teeth, her "rheumatiz" left her, she improved in condition; in fact, gained in weight and strength to such extent that she once more could bring in the coal and the kindling.

When the maid of all work visited the dentist because of toothache, she came home spitting blood, but that was all.

One by one, the decayed teeth left their moorings, and her smile became that of the family watchdog through a picket fence. Later on, she made the final visit to the dentist and shed the remainder of her grinders, appearing soon after with a temporary plate which, in turn (after the bone had filled in), yielded place to a permanent artificial denture. Then all her troubles were over, unless she married the town-sot or tied up with a four-plus Wassermann clad in trousers or a walking representative of Neisser's favorite plaything.

This little attempt at levity brings us to facts, hard, iron facts. Each year brings a new fad in medicine (and dentistry). Sometimes a thing to be promptly discarded, after having been tried in the balance, and at other times a something that shows a rift in the clouds that obscure the *ultima Thule* of the medical and dental ambition.

My earliest recollection of dentistry was the rule to extract any tooth that ached. My father was an excellent dentist, yet, despite my early training along dental lines, I hopped the dividing fence and cast my lot with the medical fraternity; "not, that I loved Caesar less, but, that I loved Rome more." I well remember the lame, the halt, and the (almost) blind coming into my father's office and departing, minus a multitude of snags and roots. I have seen the same patients come back for artificial teeth, improved in appearance and having gained in weight, despite the fact that, during the interim, they had been upon a soft diet that could be disposed of by unarmed gums. And, with subsequent mastery of the art of mastication by means of artificial teeth, said patients bloomed afresh into ruddy health. Why?

### ABSCESSED TEETH AND CHRONIC AILMENTS

Far too lengthy for the contribution that I purpose to limit to less than a folio would be the discussion of body-infections from decayed (abscessed) teeth. Consider the decay that causes an inflamed and, later, a dead and decaying nerve; and, owing to the plug of foodstuffs that effectually blocks all drainage through the crown of the tooth, the pus finds outlet through the apex of the root of the tooth, and there, in the spongy bone, it thrives and founds a numberless family, which may show a danger-signal by a "gum-boil," but, not infrequently, exhibits itself in the form of arthritis, appendicitis, endo- or pericarditis, cholecystitis and many other forms of "itis." That such things have happened, even the most conservative will admit.

And now comes the danger-line. Let him that is without error among you cast the first stone. It is here that sages will disagree. Shall the dentist endeavor to sterilize the root and save the tooth or should the tooth be extracted at once? *Quien Sabe?*

So, we must endeavor to penetrate further into the mysteries of the apex and the apical abscess. The radical dentist, by means of microscope-slides, shows that the abscessed cavity never heals completely; that a tooth in which the nerves are dead is a foreign body in the alveoli and should be removed, as becomes a foreign body. The ultraconservative dentist assures us that "thousands of these teeth have given no trouble and that crown and bridge-work are far better than clacking porcelain."

But, while I have your undivided attention, I will state that even the ultraconservative dentist, when ordering dental X-ray work, invariably (unless all teeth are to be rayed) calls attention to all crowns and bridge abutments, and desires that they be given careful attention. And there begin the troubles of the roentgenray-specialist.

### UNCERTAINTIES OF INTERPRETATION OF RADIOGRAMS

How can I prove that there is an active abscess under a given tooth? The answer is delightfully short. "I CAN'T"—that is, without the assistance of a few questions and some common sense. Unfortunately, the necromancer that works the jigger that makes the X-rays come out of the little opening is supposed to be many things that he *is NOT*. Among these things, are the roles of fortune-teller and prophet of the future. *But*—given a case wherein a "gum-boil" has caused the patient to seek dental consolation and said dentist sending the subject for a radiogram and then, when the film has been introduced into the mouth, back of the "gum-boil," showing an apical abscess; this, plus a history that the tooth "feels longer than the rest," is a fair indication of active trouble.

Were one of the readers to sit upon a 12-inch high-explosive shell and peck at the percussion-end until the shell exploded, he, too, would be convinced that the shell was active.

A man can ponder at the edge of a shell-hole and say, "A shell exploded there." If grass is growing in the cavity, he can be reasonably sure that the explosion was not a recent one. But, in any event, he can not be sure that there are no fragments of shell left buried in the crater. So with the tooth-abscess. Maybe it is ancient, perhaps fairly recent, but, we have not the knowledge whether or not the justly famous streptococcus viridans has occupied apartments in the alveoli. Most of the investigations show that Mr. and Mrs. S. Viridans are there and at home all the time.

Hence, we have a camp of physicians and dentists that say: "If it aches, have it out; if it has ached, have it out." And, we can not give a much safer answer.

It were useless for me to describe a rheumatic wrist in full bloom subsiding coincidentally with the removal of an abscessed cuspid tooth. Please, take my word for it. It were supererogation for me to state that, in all the years that I have been radiographing teeth, I never have failed to find apical abscesses in every case of "rheumatism" brought me for skiagrams of the teeth.

Let us agree that far too much is expected immediately after the removal of teeth found guilty. Were the sockets curetted or did the dentist allow the involved area to be sealed in by process of nature? In extreme instances, the deposit of the "deformans" is so great that it never can be wholly resorbed. This is our misfortune rather than our fault. The percentage of recoveries is high, much higher than before the "take the picture of my teeth" became the slogan of our rheumatics.

In these days of progress, when our advance-guards are so close upon the haunches of the enemy that our guards can count the hobnails upon said enemy's shoes, a mistake in diagnosis may be the cause of failure where dental films are called to account. I have seen a neuritis (following the influenza of recent months) so simulating the old and familiar "sciatica" that the first thought was to radiograph the teeth of the sufferer. But, when he took them into his hands and passed them to the doctor, with a friendly "Go as far as you like, but, do not break them," the doctor wisely began to search for other possible avenues of infection.

### ALWAYS HAVE THE TEETH RADIOGRAPHED

It is possible to theorize until the bottomless pit is transformed into a skating park and, yet, not reach facts. As I see the rift of light through the clouds it reads like this: "The teeth are responsible for a multitude of ailments." It is never supererogation to have them examined, although, many times, the seat of trouble will be found elsewhere.

Do not expect everything of your dentist. He will help wonderfully; still, he is not infallible. The work of dental radiography is new, so new that we do not fully comprehend everything that we see upon the plates.

In interpreting films, two things are misleading. The anterior palatine canal, if the angle of raying is not perfect, may simulate an abscess under either of the upper central teeth. Also, the shadow of the inferior mental foramen may cause like confusion, by overlying a bicuspid.

Make friends with the roentgenologist, and do not be superior to asking him for his opinion and interpretation.

(Note, kindly, that I am not speaking of the mechanical "laboratory-man," who is only a technician and who is without medical education.) The roentgenologist sees hundreds of films where you see one. "Practice makes perfect."

In conclusion, just think back to the days when artificial teeth were the rule, and go over those cases that were rheumatic, and try to recall the condition of the teeth in such cases. Mouths full of snags? Sure! How well you remember the Roquefort-cheese breath and the tongue that required an acid bath to tell whether it was coated or plated. Yet some of the advanced thinkers of those days smelled the mouse that we have visualized, but, most of them thought that the bacteria of decay were ground into the food and hence affected the digestive organs alone. They did not dream that mastication was pumping a myriad of germs from the apices of the teeth into the blood-stream.

EMPIRICISM AHEAD OF SCIENCE

I well remember my father (a dentist), twenty years ago, refusing to install bridge-work; offering as his reason the conviction that bridge-work was unsanitary, citing his experiences in removing bridges and extracting the "abutments," with a notable improvement in the health of the patients. Bringing the theme down to date, recently, a dentist in discussing the matter with me, remarked, "Your dad was twenty years ahead of his time. How we laughed at his cranky ideas then, but, we know now that he was right."

Readers, most of you will see the disappearance of bridge-work and the final obsequies of the porcelain and gold crowns mounted upon dead teeth.

Prophylaxis? Sure! Write this in letters of fire: "*Don't let your teeth ache.*" Give the same advice to your patients.—*Clinical Medicine.*

